# Parathyroid venous sampling for the preoperative localisation of parathyroid adenoma in patients with primary hyperparathyroidism

**DOI:** 10.1038/s41598-022-11238-0

**Published:** 2022-04-29

**Authors:** Joon Ho, Donggyu Kim, Ji-Eun Lee, Namki Hong, Byung Moon Kim, Dong Joon Kim, Jinkyong Kim, Cho Rok Lee, Sang-Wook Kang, Jong Ju Jeong, Kee-Hyun Nam, Woong Youn Chung, Yumie Rhee

**Affiliations:** 1 Department of Surgery, Severance Hospital, Yonsei Cancer Center, Yonsei University College of Medicine, Seoul, 03722 Republic of Korea; 2 Department of Internal Medicine, Endocrine Research Institute, Severance Hospital, Yonsei University College of Medicine, Seoul, Republic of Korea; 3 Department of Radiology, Severance Hospital, Yonsei University College of Medicine, Seoul, South Korea; 4 Department of Surgery, Yongin Severance Hospital, Yonsei University College of Medicine, Yongin, Republic of Korea

**Keywords:** Parathyroid diseases, Endocrine system and metabolic diseases

## Abstract

Preoperative localisation studies are essential for parathyroidectomy in patients with primary hyperparathyroidism. If the location of abnormal parathyroid glands cannot be identified through non-invasive studies, parathyroid venous sampling (PVS) may be employed. In this study, we evaluated the utility of preoperative PVS in parathyroid surgery. Patients with primary hyperparathyroidism who underwent preoperative PVS at Severance Hospital between January 2015 and June 2020 were identified. Patients for whom the results of non-invasive imaging studies were inconsistent or negative underwent PVS. The results of PVS were compared with operative findings and pathologic results. For 14 patients, the results of preoperative ultrasonography and ^99m^Tc-sestamibi single-photon emission computed tomography (SPECT) were negative; for 20 patients, either the result of only one test was positive, or the results of the two tests were inconsistent. With respect to the lateralisation of diseased adenoma, the results of PVS and pathological examination were inconsistent only for one patient in either group (total: 2/34 patients). This study showed that PVS could be used effectively for preoperative localisation in patients with primary hyperparathyroidism in whom the location of diseased parathyroid glands cannot be determined through non-invasive image studies.

## Introduction

Primary hyperparathyroidism (PHPT) is not a rare endocrine disease, and it has an estimated prevalence of 50 cases per 100,000 people^1–3^. In 85% of PHPT cases, the condition is caused by the presence of a single adenoma; 10–15% of PHPT cases involve multi-gland disease, and less than 1% of cases involve parathyroid cancer^4^. Surgical removal of diseased glands is employed for the curative treatment of PHPT^5^. Bilateral neck exploration (BNE) is associated with relatively long operative times and sequelae such as nerve damage; thus, focused minimally invasive parathyroidectomy is the preferred surgical approach for the treatment of PHPT. Consequently, identifying the precise location of diseased glands before the performance of parathyroidectomy is of great importance^6,7^. Non-invasive imaging studies such as ultrasonography (US) and ^99m^Tc-sestamibi single-photon emission computed tomography (SPECT) are commonly used for preoperative localisation. Although the diagnostic accuracy of these techniques is high, in some cases, the location of diseased parathyroid glands cannot be determined through these imaging studies, or the results of the studies are inconsistent^8^. In such cases, additional non-invasive imaging studies such as four dimensional-computed tomography (4D-CT), methionine positron emission tomography (PET), or fluorocholine PET are performed. However, there are certain cases in which the location of such lesions and glands cannot be confirmed through any of the aforementioned examinations; in such cases, parathyroid venous sampling (PVS) could be used for the preoperative localisation of diseased parathyroid glands. PVS has been proposed as a useful tool for patients whose preoperative non-invasive imaging is inconclusive^9–11^. Therefore, in this study, we aimed to present the diagnostic usefulness of preoperative PVS for patients with PHPT in whom the location of abnormal glands cannot be determined through non-invasive examinations.

## Results

The mean age of all patients was 58.29 years, and 29 patients (85%) were women. Depending on the results of imaging studies for the 34 included patients, the patients were divided into a negative group and a discordant group. The location of diseased glands in 14 patients could not be determined through either US or SPECT, and these patients were included in the negative group. In this group, five patients underwent methionine PET, one patient underwent fluorocholine PET, and seven patients underwent 4D-CT; however, using these imaging studies, the location of parathyroid lesions and diseased glands could not be determined in any of these cases. Among this group of patients, the final results of pathological examinations and PVS results were consistent for all patients except one. For 20 patients, either the location of diseased glands could only be determined through one of the two aforementioned tests, or the results of the two tests were inconsistent; these 20 patients were included in the discordant group. In the discordant group, one patient underwent methionine PET, and five patients underwent 4D-CT; however, using these imaging studies, the location of parathyroid lesions and diseased glands could not be determined for any of these patients. For all 20 patients in the discordant group, the results of pathological examinations and PVS were consistent for all patients except one. Among the 34 included patients, the mean preoperative PTH level was 119.73 pg/mL, and the mean intraoperative PTH-level drop rate was 74.74%. The perioperative PTH-level drop rate comparing the preoperative PTH and the PTH level ​​within 1 day after surgery was 78.09%, and the average PTH level 6 months after surgery was 39.23 pg/mL. In addition, the mean calcium level 6 months after operation was 9.21 mg/dL (Table 1). With respect to the correct lateralisation of diseased parathyroid glands, the final results of pathological examinations and PVS were consistent for 32 of the 34 patients (Table 2). Data on the negative group regarding the additional imaging studies performed, results of PVS, and results of pathological examinations are summarised in Table 3.

**Table 1:** Patient characteristics.

Parameters	Values
Age (years)	58.29 ± 16.14
Sex [no. of women (%)]	29 (85.3)
**Diseased-gland location**	
Right [frequency (%)]	19 (55.9)
Left [frequency (%)]	14 (41.2)
Bilateral [frequency (%)]	1 (2.9)
Preoperative calcium level (mg/dL)	10.75 ± 0.90
Postoperative calcium level (mg/dL)	8.57 ± 0.35
Preoperative PTH level (pg/mL)	119.73 ± 41.55
Postoperative PTH level (POD#1) (pg/mL)	22.23 ± 16.27
Perioperative PTH-level change (%)	78.09 ± 18.51
Intraoperative PTH-level change (after 10 min) (%)	74.74 ± 19.23
Miami criterion met	32/34 (94.12%)
Postoperative calcium level (POM#6) (mg/dL)	9.21 ± 0.52
Postoperative PTH level (POM#6) (pg/mL)	39.23 ± 15.18

PTH: Parathyroid hormone, POD: Post operation days, POM: Post operation months, Miami criterion: Intraoperative PTH drop ≥ 50% from the highest of either pre-incision or pre-excision level at 10 min.

**Table 2:** Diagnostic success of parathyroid venous sampling (PVS).

	Negative group (n = 14)	Discordant group (n = 20)
Direction towards right or left (%)	13/14 (92.9%)	19/20 (95.0%)
Direction towards lower or upper on the same side (%)	11/14 (78.6%)	16/20 (80.0%)

**Table 3:** Detailed description of postoperative localisation through PVS and results of pathological examinations (for the negative group).

	Diseased-gland location determined through PVS	Additional imaging studies performed	Diseased-gland location determined through pathological examination
Case 1	Rt. lower	Methionine PET	Rt. upper
Case 2	Lt. lower	4D-CT	Lt. lower
Case 3	Lt. lower	Methionine PET	Lt. lower
Case 4	Lt. lower	4D-CT	Lt. lower
Case 5	Rt. lower	4D-CT	Rt. Upper & lower
Case 6	Rt. lower	4D-CT	Lt. lower
Case 7	Rt. lower and Lt. lower	4D-CT	Rt. lower & Lt. lower
Case 8	Rt. upper	Fluorocholine PET	Rt. lower
Case 9	Lt. upper	4D-CT	Lt. upper
Case 10	Rt. upper	4D-CT	Rt. upper
Case 11	Lt. upper	Methionine PET	Lt. upper
Case 12	Lt. lower	None	Lt. lower
Case 13	Rt. lower	None	Rt. upper
Case 14	Lt. lower	Methionine PET	Lt. lower

All results of additional imaging studies were ambiguous. PVS: parathyroid venous sampling; PET: positron emission tomography; 4D-CT: four-dimensional computed tomography; Rt.: right; Lt.: left.

## Discussion

With the development of several diagnostic techniques and tools, there has been an increase in the use of minimally invasive surgery (MIS); in recent times, MIS has been performed more frequently than wide excision. Currently, various MIS procedures, such as those involving the use of laparoscopy and robotic surgical techniques, are performed by healthcare professionals; such techniques can also be used in parathyroid surgery. With respect to the treatment of patients with parathyroid lesions, in order for MIS to be possible, it is important to determine the exact location of lesions through preoperative examinations. Regarding cases of PHPT, in which surgical removal is employed for curative treatment, accurate preoperative localisation of hyperfunctioning parathyroid glands is extremely important. In such cases, if it is possible to perform MIS instead of BNE, which is time consuming, through effective preoperative localising, certain benefits could be derived by patients. BNE is inevitably associated with long operative times, extensive wounds, and complications such as nerve damage; in this regard, it has previously been reported in studies that the number of complications associated with MIS is lower than that associated with BNE^12,13^. Furthermore, compared with the incisions made in BNE, those made in MIS are smaller; thus, MIS offers a cosmetic advantage.

The most commonly used modalities for preoperative imaging in cases of PHPT are US and ^99m^Tc-sestamibi SPECT. It has previously been determined that the diagnostic accuracy of both tests is good, and according to the findings of previous studies, the sensitivity of US for the preoperative localisation of diseased parathyroid glands is between 70 and 90%, and the sensitivity of ^99m^Tc-sestamibi SPECT is approximately 80%^8,14,15^. However, in several cases involving small or multiple parathyroid lesions, the location of lesions cannot be determined using these imaging techniques. In such cases, other imaging techniques such as 4D-CT and PET can be performed, and the excellent diagnostic accuracy of these techniques have been revealed in certain studies^16–18^. However, some tests are not cost-effective, and depending on the size of a medical institution or health facility, the implementation of such tests may be difficult or require a long waiting period. Moreover, in certain cases, the location of lesions cannot be confirmed even after additional imaging tests have been performed; thus, for preoperative localisation, additional means are required.

Several studies have reported the efficacy of FNAB for parathyroid glands. Erbil et al. reported that the positive predictive value and sensitivity of PTH testing with FNAB to localize parathyroid adenoma were 100%^19^. Abraham et al. found that the sensitivity of cytological evaluation was 91% with a specificity of 95%^20^. Conversely, some studies have shown that FNA in parathyroid neoplasms increases the risk of massive hemorrhage, tumor seeding, parathyromatosis, or recurrence^21,22^. In addition, several case reports reported complications such as bleeding after FNAB and airway obstruction that may occur in more severe cases^23,24^. As such, FNAB of the parathyroid gland has not been generally recommended and its use is controversial. According to the guidelines of the American Association of Endocrine Surgeons, preoperative parathyroid FNA is not recommended, except in unusual and difficult cases of primary hyperparathyroidism, and should not be performed if parathyroid carcinoma is suspected^25,26^.

The purpose of this study was to confirm the diagnostic value of PVS in cases of PHPT in which the location of diseased parathyroid glands could not be determined through various preoperative imaging studies; furthermore, we endeavoured to confirm whether PVS could be used as a second-line diagnostic modality in such cases. Considering the aforementioned limitations associated with the use of 4D-CT, methionine PET, or fluorocholine PET, the role of PVS as a second-line evaluation tool can be considered.

The diagnostic value of PVS has been analysed in previous studies. Barczynski et al. reported that among 50 patients who had a solitary parathyroid adenoma, true-positive results for the detection of the adenomas could be obtained for 33.33% (8/24) of patients who underwent only US and 65.4% (17/26) of patients who underwent US combined with bilateral internal jugular venous sampling with rapid PTH assay^27^. It was further confirmed in two other studies that selective venous sampling enables the localisation of parathyroid lesions in patients for whom the results on non-invasive imaging studies are negative, equivocal, or discordant^28,29^. In the present study, we found that among 41 cases of PHPT(34 included cases + 7 cases of PVS values less than 1.5 times that of peripheral blood), in which either preoperative localisation could not be achieved through US and SPECT, or the results of these imaging studies were inconsistent, the sensitivity of PVS for the localisation of diseased parathyroid glands was 78%. Additionally, among the 34 patients who were ultimately considered, lateralisation through PVS was possible for 32 patients, which demonstrates the effectiveness of PVS as a diagnostic tool.

Among the study participants, 22 patients had parathyroid adenoma, whereas 12 had pathologic diagnosis of parathyroid hyperplasia. After removal of culprit lesion in those 12 participants, they had successful recovery of PTH after 6 months of surgery, without evidence of persistent or recurred hyperparathyroidism, which followed the clinical course of single gland disease rather than multi gland disease. We did not find any evidence of familial diseases in these participants. Although the possibility of recurrence cannot be totally excluded in these participants with pathologic diagnosis of hyperplasia, these findings still support the value of parathyroid venous sampling in localizing culprit lesions in difficult cases of primary hyperparathyroidism.

Complications such as bleeding, infection, pseudoaneurysms, arteriovenous fistulae, and hypersensitivity to contrast agents may occur as a result of PVS. However, these complications rarely occur, and none of the patients included in this study experienced any complications. Compared with the risks associated with BNE, those associated with PVS are minor and rare. Exposure to radiation during PVS is another risk that must be considered. With respect to the patients considered in this study, the median duration of PVS (according to our protocol) was about 20 min (interquartile range: 16–24 min), and the dose of radiation, which the patients were exposed to, ranged from 1.26 mSv to 5.3 mSv. These doses ​​are similar to those reported in previous studies, and are only about half the exposure dose of 4D-CT (10.4 mSv)^30,31^. Even so, PVS is not the first technique performed in cases of PHPT, and it may be used when the location of diseased glands cannot be determined through non-invasive imaging studies^25^. Although PVS is an invasive technique that involves exposure to radiation, it is a method that can be considered for the localisation of diseased parathyroid glands because through its use, the repetitive performance of surgery can be avoided, and the use of MIS can be enabled.

This study has certain limitations. First, the study has a retrospective design, and the number of patients considered was small. However, all patients who were diagnosed with PHPT and underwent surgery at a single institution during the specified time period were initially considered; among these patients, data on a smaller group of patients who underwent PVS were finally selected and their data were analysed. Second, only patients who underwent PVS were included, and we did not consider the surgical or perioperative outcomes in patients who underwent BNE but not PVS. Since patients who underwent parathyroidectomy at a single institution were regarded in this study, it is impossible to apply the selective selection that reflects the research purpose in the selection of diagnostic tests. This is because it can affect the surgical method or surgical outcome for each patient. This can be analysed in future multi-centre studies. Third, it was implemented with conventional PVS(cPVS). Various studies have reported on the higher diagnostic utility of super-selective PVS(sPVS)^29,32–35^.

In the case of cPVS, PTH is measured at a total of 8 points including the upper, mid, and lower points of the internal jugular vein on both sides and two points on the brachiocephalic vein on both sides. However, in the case of sPVS, PTH is additionally measured in the superior, middle, and inferior thyroid veins on both sides. In addition, when the main inferior thyroid trunk is present, PTH is also measured in the thymic and superior intercostal veins, and PTH is measured in at least 15 groups to more accurately evaluate the location of the patholic parathyroid^34^. Among the patients included in this study, the case of two patients who failed lateralization is likely due to the presence of the common trunk of the inferior thyroid vein. Therefore, if more accurate results were derived by conducting sPVS in this study, more advanced research results could have been obtained. However, the institution is in the beginning stage of PVS, and cPVS is implemented due to various circumstances, so the application was somewhat limited. Because cPVS is performed under these limited conditions, thyroid vein anatomy and the presence of common trunk should be considered, and more attention should be paid to interpretation of the results of inferior sites. On the other hand, this study is considered to have the advantage of being able to proceed with focused parathyroidectomy rather than BNE surgery through at least lateralization in patients with negative and discordant results on non-invasive imaging studies. In the further, we plan to conduct sPVS in the future and proceed with advanced research based on the results.

## Methods

**Figure 1:**
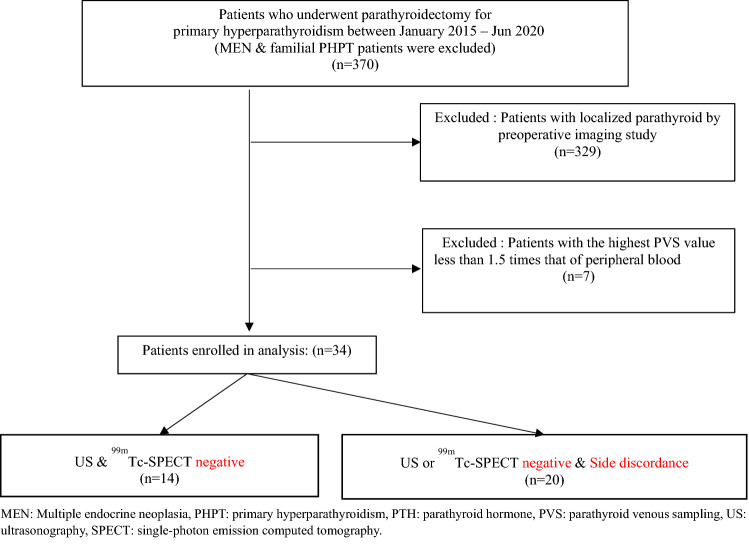
Flow-chart describing the inclusion of patients and the study population; MEN: Multiple endocrine neoplasia, PHPT: primary hyperparathyroidism, PTH: parathyroid hormone, PVS: parathyroid venous sampling, US: ultrasonography, SPECT: single-photon emission computed tomography.

The medical records of patients who were diagnosed as having PHPT and underwent focused parathyroidectomy at Yonsei University Hospital between January 2015 to June 2020 were extracted and retrospectively analysed. During the medical records review, MEN and familial PHPT patients were first excluded. For the preoperative localisation of diseased parathyroid glands, all of these patients had undergone non-invasive imaging examinations. For all patients, US and ^99m^Tc-sestamibi SPECT were performed first; if the location of diseased glands could not be determined through these techniques, additional examinations such as 4D-CT, methionine PET, and fluorocholine PET were performed. PVS was then performed for patients in whom the location of diseased glands could not be confirmed even through the aforementioned additional examinations; a parathyroid hormone (PTH) level determined through PVS in a patient that was ≥ 1.5 times the level of PTH in the patient’s peripheral blood was considered a meaningful and significantly high level^36^. A total of 370 patients were identified, and 329 patients in whom the location of diseased glands had been confirmed through preoperative imaging studies were excluded. Seven patients in whom the highest PTH level determined through PVS was < 1.5 times the level of PTH in their peripheral blood (n = 7) were excluded; consequently, 34 patients were finally included, and their data was analyzed (Fig. 1).

### At our institution, two dedicated radiologists with experiences performed the procedure

After local anesthesia with 1% lidocaine, venous access was acquired via the right femoral vein using Seldinger method with the insertion of four-French sheath, four-French nontapered catheter, and 035 Terumo guidewire. During PVS, blood samples were obtained from eight predetermined venous locations in the upper (above the cervical vertebrae, at the C3 level), middle (between levels C3 and C5), and lower (between levels C5 and C7) segments of the bilateral internal jugular veins (IJV) and from the bilateral brachiocephalic veins (below level C7, at the point of convergence of the IJV and subclavian vein). Peripheral blood samples were collected directly from the femoral vein (located in the femoral sheath). For localisation, the maximum PTH-gradient value was considered the PTH_max_ value^36^.

Each patient underwent intraoperative PTH monitoring, and PTH was confirmed by evaluating blood samples obtained (through an arterial line inserted into the radial artery) 5 and 10 min after each specimen had been delivered. For each patient, the operation was terminated when the patient’s PTH level was found to be ≥ 50% lower than the baseline level according to Miami criteria (the PTH level measured on the day before operation)^37,38^.

This study was approved by the Institutional Review Board (IRB) of Severance Hospital (4–2021-0691), and conducted in accordance with the principles of the Declaration of Helsinki. The IRB waived the requirement for obtaining informed consent due the study’s retrospective design.

## Conclusions

PVS may have certain limitations, but it is an exact technique for the preoperative localisation of parathyroid lesions and diseased parathyroid glands whose locations cannot be determined through non-invasive imaging studies. Therefore, PVS may be a sufficient complementary means when the location of the diseased parathyroid cannot be confirmed by imaging tests such as US, SPECT, 4D-CT, and PET. Consequently, with the use of PVS, it is possible to eliminate the need to perform operative bilateral neck exploration and enable the use of MIS, which is associated with less invasiveness to patients.

## Data Availability

The datasets used and/or analysed during the current study available from the corresponding author on reasonable request.
